# Utilizing Freeze-Thaw-Ultrasonication to Prepare Mesoporous Silica-Encapsulated Colloidal Silver Nanoaggregates with Long-Term Surface-Enhanced Raman Spectroscopy Activity

**DOI:** 10.3390/s25061840

**Published:** 2025-03-15

**Authors:** Shuoyang Yan, Ling Chen, Zhiyang Zhang

**Affiliations:** 1 School of Materials Science and Engineering, University of Jinan, Jinan 250022, China; ysyang026@163.com; 2 Engineering Research Center, Shandong Key Laboratory of Coastal Environmental Processes, CAS Key Laboratory of Coastal Environmental Processes and Ecological Remediation, Yantai Institute of Coastal Zone Research, Chinese Academy of Sciences, Yantai 264003, China

**Keywords:** surface-enhanced Raman spectroscopy, SERS substrate, silver nanoclusters, mesoporous silica coating, stability

## Abstract

Surface-enhanced Raman spectroscopy (SERS) is widely employed due to its high sensitivity and distinctive fingerprinting capabilities. Colloidal nanoaggregates are commonly used as SERS substrates because of their mobility and the abundance of “hotspots”. Although the reagent-free “freeze-thaw-ultrasonication” method for preparing Ag nanoaggregates (AgNAs) does not introduce additional background interference and maintains the original interfacial properties of AgNAs, their unstable physical nanostructure limits SERS detection to just 7 days. Herein, we demonstrate mesoporous silica-encapsulated colloidal Ag nanoaggregates (AgNAs@m-SiO_2_) by combining a freeze-thaw-ultrasonication method and a cetyltrimethylammonium bromide (CTAB)-assisted silanization reaction, achieving long-term SERS stability of more than two months. The prepared AgNAs@m-SiO_2_ serve a dual capability: (1) preserving electromagnetic “hotspots” for ultra-sensitive detection (e.g., malachite green detection limit: 3.60 × 10^−^^8^ M), and (2) maintaining structural stability under harsh conditions. The AgNAs@m-SiO_2_ substrate exhibited superior structural stability after 50 min of ultrasonic treatment, with an initial SERS signal retention of 91.8%, which is twice that of the bare AgNAs (retention of 45%). The long-term performance further highlighted its superiority: after 70 days of storage, the composite maintained 84.3% of its original signal strength, outperforming the uncoated controls by over ten times (which retained only 8%). Crucially, the substrate’s robust design enables the direct detection of contaminants in real environmental matrices (river and seawater) for qualitative analyses and water quality assessments, thus validating its suitability for environmental sensing applications in the field.

## 1. Introduction

Surface-enhanced Raman spectroscopy (SERS) has been widely used in the fields of chemical analysis, environmental monitoring, and biomedicine due to its high sensitivity and characteristic fingerprint identification [1,2,3,4]. The signal enhancement of SERS originates from a combination of electromagnetic field enhancement and chemical mechanisms, in which the electromagnetic field enhancement mechanism plays a dominant role, especially the strong electromagnetic field formed in the nanogap region of the “hotspot”, which plays a key role in the enhancement of the Raman signal [5,6,7,8]. These critical hotspots predominantly exist in nanoparticle aggregates, making aggregated nanostructures quintessential SERS substrates. Contemporary aggregated SERS substrates fall into two main categories: solid-supported assemblies and colloidal aggregates. Solid-phase substrates are usually made by immobilizing silver or gold nanoparticles on rigid surfaces (e.g., silicon wafers, glass slides) [9,10], which achieve high hotspot densities through the stacking of nanoparticles and provide better sensitivity [11]. However, their practical application faces inherent limitations, including the coffee-ring effect during solvent evaporation, which leads to inhomogeneous distribution and poor reproducibility [12,13]. On the contrary, colloidal nanoaggregates maintain the dynamic assembly of nanoparticles in liquid suspensions with fluidity. This fluidic property not only enhances the interaction between the electrolyte-free substance and the hotspot but also reduces signal fluctuations due to focusing errors during optical measurements [14,15]. Due to these advantages, colloidal aggregates have emerged as key SERS substrates [7,8]; for example, by combining microfluidics with colloidal metal nanoaggregates, researchers can achieve high-throughput on-line SERS assays that enable rapid identification and quantification of a range of samples [16]. A common strategy for preparing colloidal metal nanoaggregates involves inducing the aggregation/assembly of monodispersed nanoparticles [17,18,19,20]. These methods usually require the addition of chemical agents (e.g., organic ligands or high-concentration salts) to initiate aggregation. However, the introduction of organic ligands may add additional background signals, alter the surface adsorption capacity (e.g., by changing the surface charge) [21], and affect the SERS performance. Salt-induced aggregation is facile and cost-effective, but the resulting colloidal nanoaggregates typically exhibit short-term stability [22,23]. To address these limitations, our team developed a “freeze-thaw-ultrasonication” method to prepare stable colloidal silver nanoaggregates (AgNAs) [14]. Unlike traditional organic ligands or salt-based approaches, this method avoids introducing any chemical reagents, thereby preserving the chemical interface properties of nanoparticles and minimizing background interference. However, despite their advantages, AgNAs prepared via this method remain susceptible to environmental perturbations, and the AgNAs can only maintain a stable SERS activity within 7 days due to the disassembly of the nanoaggregate over time [14]. Therefore, it is essential to prepare colloidal AgNAs with long-term SERS activity.

Efforts to enhance the structural stability of substrates have primarily focused on surface modification strategies, notably through small-molecule ligand engineering or inorganic encapsulation techniques, such as silica (SiO_2_) coatings [18,21,24]. Ligand-based approaches, exemplified by thiolated polymers (e.g., polyethylene glycol, PEG) or alkanethiol self-assembled monolayers, leverage strong chemisorption bonds to sterically stabilize nanostructures [25]. Although these modifications are effective in suppressing nanoparticle aggregation, they can affect the analytical performance due to the additional background signal caused by the ligand’s own SERS signal. Inorganic coatings are more suitable for the structural stability of SERS substrates as opposed to the structural stability of nanoparticles stabilized by organic ligands, which do not cause extra background signals. Inorganic coatings, particularly silicon shells, offer an alternative route by physically isolating plasmonic nanostructures from environmental perturbations. SiO_2_ can effectively protect the nanostructures, but the traditional SiO_2_ coating strategy often hinders the diffusion of analytes into the “hotspot” region due to the large thickness and denseness of the shell layer, resulting in low sensitivity [26,27,28]. Achieving a balance between stability and sensitivity in SERS substrate design remains an important challenge.

To solve this problem, herein, we demonstrate a mesoporous silica (m-SiO_2_)-encapsulated colloidal Ag nanoaggregates (AgNAs@m-SiO_2_) by combining a freeze-thaw-ultrasonication method and a cetyltrimethylammonium bromide (CTAB)-assisted silanization reaction, achieving a long-term SERS stability of more than two months. This method not only prevents further aggregation and structural disintegration due to external perturbation through the silica skeleton but also facilitates the diffusion of the target molecules into the “hotspot” region, thus enhancing the stability and preserving the amplification effect of the SERS signal to the maximum extent. The experimental results show that the composites can significantly improve the structural stability of the substrate and exhibit excellent signal retention under long-term storage conditions, providing a new technological pathway for high-sensitivity and reproducible SERS sensing applications.

## 2. Materials and Methods

### 2.1. Chemicals and Instruments

Silver nitrate (AgNO_3_), sodium hydroxide (NaOH), malachite green (MG), crystal violet (CV), glycerol, tetraethyl orthosilicate (TEOS), and anhydrous ethanol were purchased from Sinopharm Chemical Reagent Co., Ltd., Shanghai, China, sodium citrate and cetyltrimethylammonium chloride (CTAB) were purchased from Aladdin Reagent Co., Ltd., Shanghai, China, the glass capillary tubes (with an inner diameter of 0.9–1.1 mm) were produced by the Instrument Factory of West China Medical University, Sichuan, China. Deionized water (18.2 MΩ cm^−1^) was used for instrument cleaning and sample dilution.

The UV–Vis–NIR absorption spectra were recorded on a NanoDrop 2000/2000C spectrophotometer (Thermo Scientific, Waltham, MA, USA). The SERS spectra were recorded using a DXR Raman microscope (Thermo Scientific, Waltham, MA, USA). A 532 nm laser was focused using a 5× microscope objective on the sample solution. The laser power was set to 1.25 mW, and the acquisition time for each spectrum was 1 s. The zeta potential and dynamic light scattering (DLS) size were measured on a Zetasizer NanoZS90 (Malvern Instruments, Malvern, UK). Transmission electron microscopy (TEM) images were obtained on a Talos F200XG2 (Thermo Scientific, Waltham, MA, USA). Other laboratory instruments used were as follows: a collector-type constant temperature heating magnetic stirrer (DF-101Z) from L Zhengzhou Great Wall Science and Trade Co., Ltd., Zhengzhou, Henan, China; an electronic balance and an oven from Sartorius Scientific Instruments GmbH, Göttingen, Germany; an Avinity ultrapure water machine from Beijing Zeping Technology Agency, Beijing, China; a medical centrifuge from Hunan Xiangyi Laboratory Instrument Development Co., Ltd., Changsha, Hunan, China; Ultrasonic cleaning machine (KQ3200) from Kunshan Ultrasonic Instrument Co., Ltd., Kunshan, Jiangsu, China; and a refrigerator from Anhui Kangjia Tongchuang Electrical Appliance Co., Ltd., Chuzhou, Anhui, China. A three-necked flask, a beaker, and a thermometer were purchased from local suppliers. More details can be found in the Supporting Information.

### 2.2. Preparation of Materials

Firstly, AgNAs were prepared by the “freeze-thaw-ultrasonication” method. Specifically, H_2_O (250 mL) containing 1 mL of glycerol was heated to a slight boil under vigorous stirring, and then 1 mL of AgNO_3_ (45 mg/mL) and 5 mL of sodium citrate (1% *w*/*w*) were added. After 30 min of the reaction, the color of the solution changed to greenish brown, and monodisperse silver nanoparticles (AgNPs) were obtained. They were concentrated to 25 mL by centrifugation and then frozen at −20 °C for 4 h. After thawing at room temperature, AgNAs of appropriate size and uniformity were obtained by ultrasonication for 10 min.

Then, 120 mL of H_2_O solution containing 2.5 g of CTAB and 3 mL of NaOH (0.5 M) was heated to 80 °C, and the reaction was stirred for 15 min. Then, 125 mL of the AgNA solution (the original concentrated solution was taken as 12.5 mL and diluted 10-fold) was added and stirred for 10 min. Then, 1.5 mL of TEOS and 3.25 mL of ethanol were added and reacted for 1 h, and, finally, the solution was centrifugally washed 3 times and concentrated to 10 mL to obtain AgNAs@m-SiO_2_.

### 2.3. FDTD Simulation

Three-dimensional finite-difference time-domain (FDTD) simulations were conducted using commercial software (Lumerical 2020 R2.4) to quantitatively analyze the electromagnetic field enhancement characteristics of AgNPs, AgNAs, and AgNAs@m-SiO_2_ architectures.

A linearly polarized plane wave source with a wavelength of 532 nm was propagated along the z-axis, with the electric field polarization in the x-direction, to illuminate the simulation domain. Perfectly matched layer (PML) boundary conditions were applied on all sides of the domain to minimize the artificial reflections, and a mesh overlay with a resolution of 1 nm was used around the nanoparticles to accurately capture near-field gradients. AgNPs were simulated with a diameter of 80 nm. AgNAs were modeled as AgNP dimers to simulate the local electric field at the interstitial “hotspot”. AgNAs@m-SiO_2_ was simulated with a 13 nm thick SiO_2_ shell surrounding the AgNAs.

### 2.4. SERS Detection

A total of 27 μL of AgNAs@m-SiO_2_ was mixed with 3 μL of the material to be tested to obtain 30 μL of the solution to be tested. The mixed liquid was inhaled through a capillary tube, and the SERS determination was carried out using a DXR microconfocal Raman spectrometer under the following conditions: an integration time of 1 s, a 532 nm excitation wavelength laser source with a power of 1.25 mW, and five cumulative scans.

### 2.5. Structural Stability Assessment

Aliquots were collected at defined time intervals (0, 5, 10, 15, 30, and 50 min) and mixed with an MG solution (1:10 *v*/*v* ratio, final concentration 10^−5^ M). After 10 min of incubation, SERS measurements were performed using a 532 nm laser (integration time of 1 s, 1.25 mW power, five cumulative scans), and five samples were collected from each sample.

### 2.6. Storage Stability Evaluation

The freshly prepared AgNAs and AgNAs@m-SiO_2_ suspensions were stored in amber glass vials under ambient conditions. UV-Vis absorption spectra (300–800 nm range) were recorded on day 1 and day 70 using matched quartz cuvettes. For the SERS characterization, the stored samples were mixed and incubated with a 10^−4^ M 4-mercaptopyridine (4-MPY) solution (1:10 *v*/*v* ratio), followed by 10-minute interfacial adsorption prior to spectral collection. All measurements employed identical instrument parameters to ensure comparative consistency.

### 2.7. Real Sample Detection Protocol

Spiked samples containing 10^−5^ M of MG were obtained by mixing ambient water samples (seawater from the East Gate of Yantai University and freshwater from the Fenghuangshan Reservoir) with MG. The contaminated solution was mixed with the AgNAs@m-SiO_2_ suspension (1:10 *v*/*v*) and incubated for 15 min. The mixture was aspirated using a capillary tube, and spectra were immediately collected under standardized measurement conditions.

## 3. Results

### 3.1. Materials Preparation and Characterization

**Figure 1:**
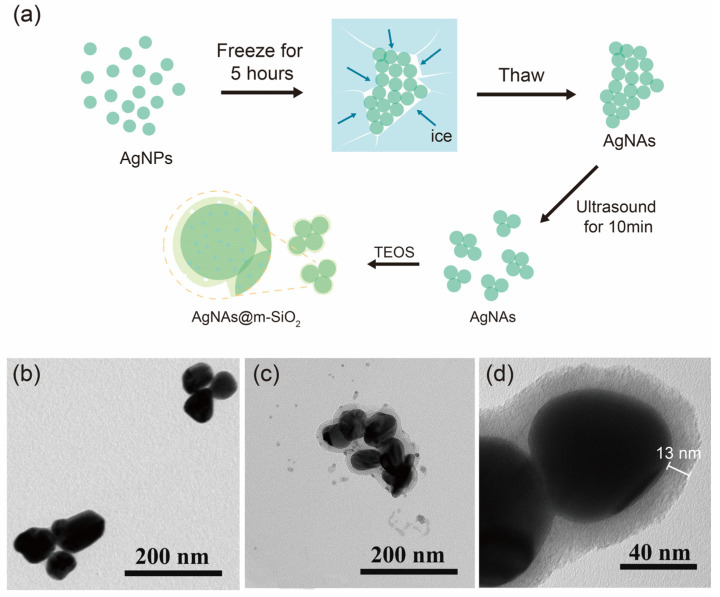
(**a**) Schematic of the AgNAs@m-SiO_2_ material preparation. (**b**) TEM image of AgNAs. (**c**) TEM image of AgNAs@m-SiO_2_. (**d**) TEM image of AgNAs@m-SiO_2_ at higher magnification.

The hierarchical synthesis strategy (Figure 1a) utilized a two-stage assembly mechanism to achieve structurally stable SERS-active nanocomposites. Firstly, AgNP aggregation was induced by a freeze-thaw-sonication method [14]. During the freezing process (−20 °C, 4 h), the significant reduction in liquid water formed a localized region of high concentration, which overcame the electrostatic repulsive force between the AgNPs capped by the citrate (zeta potential = −32.4 mV), thus triggering a torus stabilization process leading to nanoparticle aggregation [29]. The AgNAs synthesized by a low-temperature induction were relatively large (Figure S1a), which significantly compromised their colloidal stability and suspension performance in the capillary systems. Subsequently, the thawed and sunken large-sized AgNAs were sonicated. The mechanism of sonication depolymerization is that ultrasound waves propagate into the liquid medium and generate high mechanical pressure between the particles to separate them from each other. With the increasing sonication time, the average size of colloidal AgNAs gradually decreases during the sonication depolymerization (Figure S1b); the sonication time (10 min) was optimized in our previous work [14].

The second stage of m-SiO_2_ encapsulation was performed by CTAB micelles first adsorbed onto the AgNA surface by electrostatic attraction to form a cationic molecular template. The TEOS precursor then undergoes base-catalyzed hydrolysis at the liquid−solid interface, with silicate species preferentially condensing around the CTAB micelles. This results in the formation of mesoporous structures (Figure 1d) while also preserving the nanogap between neighboring particles. Transmission electron microscopy characterization confirmed that AgNAs@m-SiO_2_ exhibited a well-defined core-shell structure with a uniform mesoporous morphology (Figure 1c,d). The silica shell, approximately 13 nm thick, appeared as a relatively continuous but non-dense layer, suggesting the presence of interconnected mesopores. This porous nature contrasts with the morphology of AgNAs (Figure 1b), indicating the successful encapsulation of m-SiO_2_ while preserving pathways for molecular diffusion and accessibility to the plasmonic core. The size of AgNAs@m-SiO_2_ obtained from the DLS dynamic light scattering measurements was approximately 843 nm (Figures S1c and S2).

**Figure 2:**
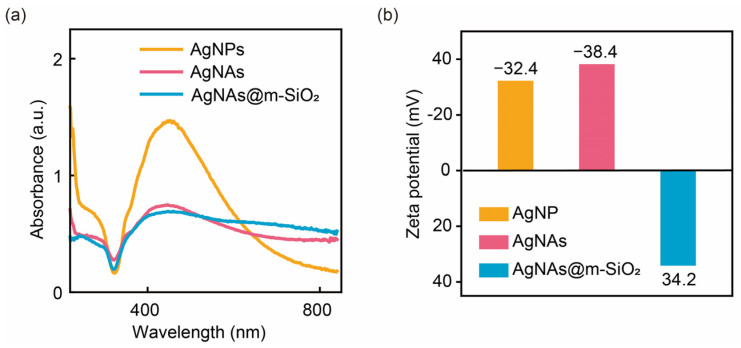
(**a**) UV–Vis absorption spectra and (**b**) zeta potentials of AgNPs, AgNAs, and AgNAs@m-SiO_2_ materials.

The optical and interfacial characteristics of the colloidal systems were systematically investigated through UV–Vis spectroscopy and a zeta potential analysis, as demonstrated in Figure 2. Monodisperse AgNPs stabilized by citrate ligands exhibit a characteristic localized surface plasmon resonance (LSPR) peak at 450 nm (yellow curve, Figure 2a), corresponding to dipolar oscillations of conduction electrons in individual 80 nm nanoparticles [30,31]. Upon the freeze-thaw-ultrasonication treatment, the LSPR profile undergoes distinct modifications: the peak intensity at 450 nm decreases with a broad absorption (600 to 800 nm). This optical transition arises from the phenomenon of longitudinal plasmonic coupling present in the aggregated AgNAs: closely spaced nanoparticles generate longitudinal plasmonic modes along the aggregation axis, resulting in a red-shifted absorption above 600 nm [32]. Moreover, the m-SiO_2_ capping method maintains the adjacent particle gap critical for SERS activity hotspots, while the mesoporous shell thickness (13 nm) remains sub-wavelength with respect to visible light (400–800 nm), minimizing the optical shielding effects. The zeta potential analysis showed that the zeta potentials of AgNPs and AgNAs are −32.4 and −38.4 mV, respectively. This is because the “freeze-thaw-ultrasonication” method did not change the surface molecular structures and, thus, can maintain its surface charging properties. In contrast, after the coating of m-SiO_2_, the zeta potential of the AgNAs@m-SiO_2_ was changed into +34.2 mV. The reason is that the formation of m-SiO_2_ needs the presence of CTAB surfactant, and the positively charged surface is attributed to the wrapped CTAB on the AgNAs@m-SiO_2_. All the results imply that m-SiO_2_ was successful and was coated on the AgNAs, and the surface charges of the nanoaggregates are modified by the CTAB.

**Figure 3:**
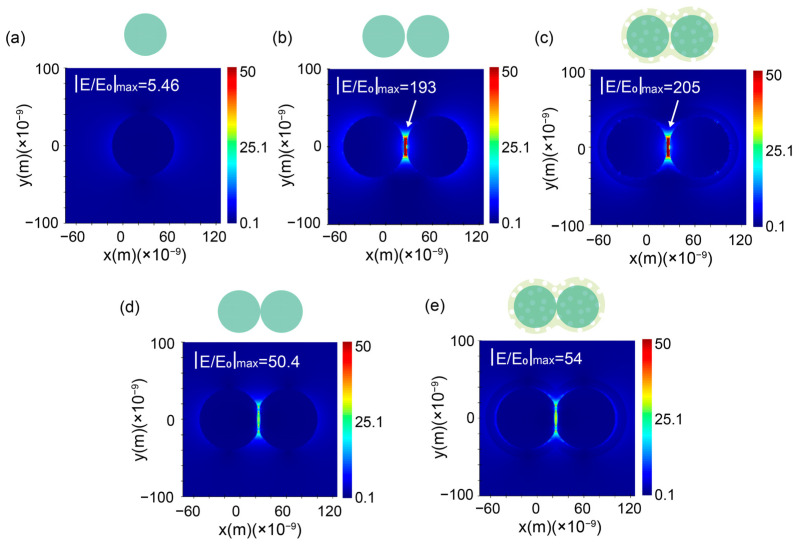
FDTD-simulated electric field distributions. (**a**) Single AgNP (80 nm): |E/E_0_|_max_ = 5.4. (**b**) AgNAs dimer (2 nm gap): |E/E_0_|_max_ = 193 (gap coupling). (**c**) AgNAs@m-SiO_2_ (13 nm shell): |E/E_0_|_max_ = 205. Simulation of contacting particles under the same conditions (**d**) AgNAs: |E/E_0_|_max_ = 50.4 (**e**) AgNAs@m-SiO_2_ (13 nm shell): |E/E_0_|_max_ = 54.

To quantify the electromagnetic enhancement characteristics of the nanostructures, three-dimensional finite-difference time-domain (FDTD) simulations were performed under 532 nm excitation. For isolated 80 nm AgNPs, the local electric field enhancement (|E/E_0_|_max_) peaked at 5.4 near the nanoparticle poles (Figure 3a), consistent with the dipolar plasmon resonance. In contrast, the AgNAs dimer (interparticle gap = 2 nm) exhibited intense near-field coupling, generating a hotspot with |E/E_0_|_max_ = 193 in the junction region (Figure 3b), and the enhancement of the “hotspot” amplifies the field by more than 35 times compared to that of a single nanoparticle. Remarkably, the AgNAs@m-SiO_2_ system retained a comparable enhancement (|E/E_0_|_max_ = 205) despite the 13 nm silica shell, corresponding to a theoretical SERS enhancement factor of 1.77 × 10^9^ (|E/E_0_|_max_^4^). As observed in the TEM image (Figure S1c), in addition to the hotspot enhancement in the interstitial spaces between particles, a “V-shaped” hotspot is also formed at the points of particle contact. To further investigate this effect, we simulated the “V-shaped” hotspot under identical conditions. Although the field strength of the “V-shaped” hotspot is lower than that of the interstitial hotspot, it still exhibits a notable enhancement effect, with a maximum field intensity of |E/E_0_|_max_ = 50 (Figure 3d). Furthermore, the corresponding enhancement factor at the “V-shaped” hotspot reaches 8.5 × 10^6^ (|E/E_0_|_max_^4^). Similar to the gap-augmented structure, the presence of the silica shell did not diminish the enhancement effect of the “V-shaped” structure (Figure 3e). This indicates that the theoretical enhancement effect after cladding the m-SiO_2_ shell (~13 nm) is consistent with the AgNAs enhancement, confirming that the mesoporous shell maintains the electromagnetic enhancement feature while adding protection.

### 3.2. Stability of Physical Structures

**Figure 4:**
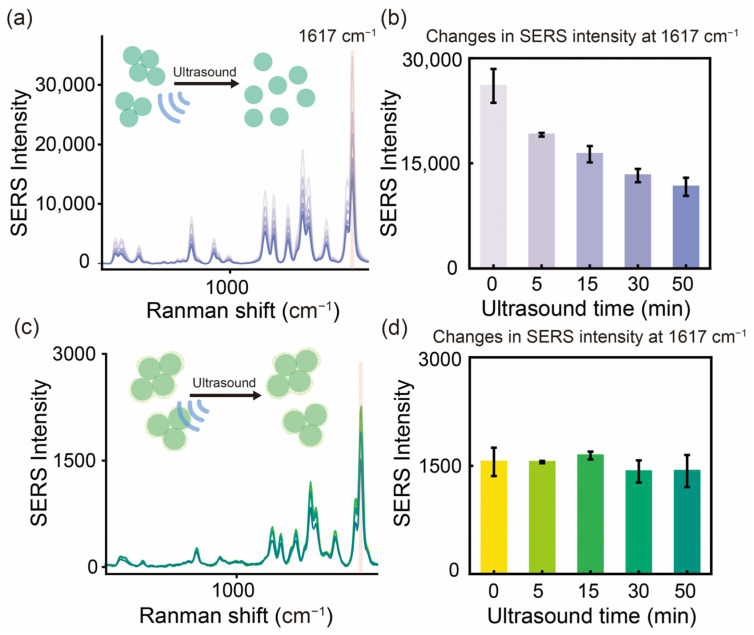
Effect of ultrasonic treatment (50 min) on the stability of the physical structure of SERS substrates. (**a**) SERS signal changes of AgNAs and (**b**) histogram of signal changes of MG (10^−5^ M) characteristic peak 1617 cm^−1^. (**c**) SERS signal change of AgNAs@m-SiO_2_ and (**d**) histogram of signal change of MG characteristic peak 1617 cm^−1^.

To assess the structural and physical stability of AgNAs@m-SiO_2_ under extreme mechanical stress conditions, progressive sonication experiments were conducted on both uncoated AgNAs and their m-SiO_2_-coated counterparts. The temporal evolution of the SERS intensity over the characteristic band 1617 cm^−1^ reveals different stabilization mechanisms between the two materials (Figure 4).

For the uncoated AgNAs, the SERS signal intensity of MG gradually decreased with the increasing sonication time (Figure 4a). Within the first 5 min, the SERS intensity dropped by 27%, primarily due to the decrease in the density of “hotspots” caused by the destruction of weakly coupled nanoaggregate particles. The trend of signal decrease then slowed down with time, and, finally, the signal decreased by about 55% at 50 min (Figure 4b), indicating a significant structural dissociation of the AgNAs (Figure 4a). In contrast, AgNAs@m-SiO_2_ showed superb stability under the same conditions (Figure 4c,d). The stable support structure formed by the m-SiO_2_ cladding enabled AgNAs@m-SiO_2_ to maintain 91.8% of the initial SERS intensity after 50 min of sonication. Notably, the characteristic Raman peaks of MG at (list key Raman shifts, e.g., 1176 cm^−^^1^, 1617 cm^−^^1^) remained nearly unchanged in intensity and spectral position, confirming that the molecular adsorption environment and electromagnetic enhancement were preserved. These results clearly indicate that the coating of m-SiO_2_ can significantly enhance the physical structure stability of the nanoaggregates, which will help the application of the colloidal SERS substrate in harsh conditions and the storage of a standard analytical reagent.

### 3.3. Long-Term Storage Stability

**Figure 5:**
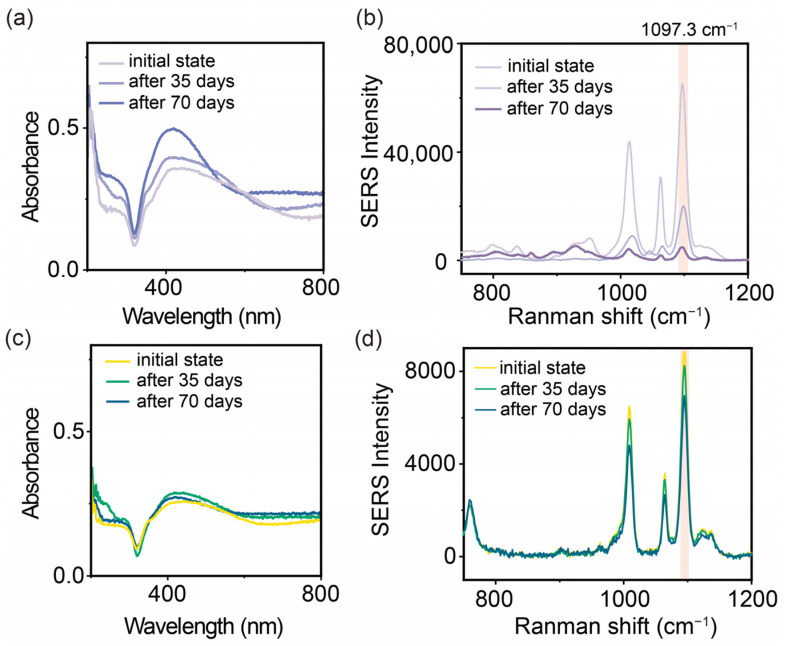
Effect of long-term storage (70 days) on the stability of SERS substrates. (**a**) Changes in UV–Vis absorption spectra and (**b**) SERS detection signals on 4-MPY (10^−5^ M) for AgNAs. (**c**) Changes in UV–Vis absorption spectra and (**d**) SERS detection signals on 4-MPY for AgNAs@mSiO_2_.

Long-term stability is considered crucial for nanomaterial-based analytical platforms, as structural instability under operational conditions can significantly impact their sensitivity and reliability. To evaluate the stability of the AgNAs@m-SiO_2_, a long-term storage experiment was conducted by comparing the SERS activity before and after 70 days of storage (Figure 5).

**Figure 6:**
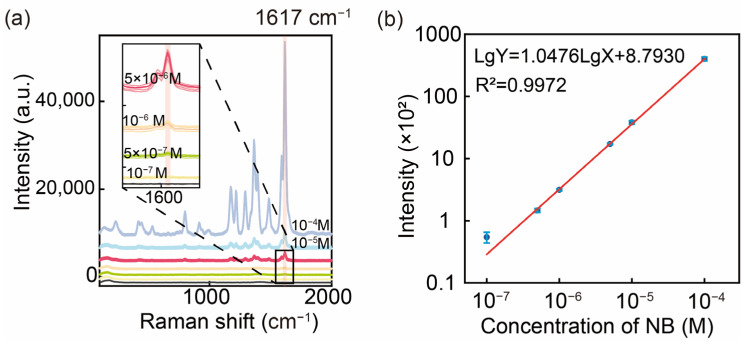
(**a**) SERS spectra of MG at different concentrations (10^−4^ M~10^−7^ M). (**b**) Log-linear plot of SERS signal intensity versus MG concentration at 1617 cm^−1^.

As a control, we first investigated the optical response of AuNAs before and after storage for 70 days. As shown in Figure 6a, during 70 days of storage, we can see a significant narrowing of the plasmon band of the AgNAs along with the increase in the plasmon band at 410 nm (a characteristic band of monodisperse AgNPs). This narrowing plasmon band reflects the disassembly of the AgNAs. Subsequently, the effect of the storage on the SERS activity was further investigated by using the model reporter (4-MPY). After 70 days of storage, the uncoated AgNAs showed a significant decrease (decreased by 92%) in the SERS signal, and only 8% of the original intensity was retained (Figure 5b). In contrast, the AgNAs@m-SiO_2_ showed a tiny change in the UV absorption spectra before and after storage (Figure 5c), indicating that a little change in the physical structure of the nanoaggregates had occurred. Consistently, the SERS signal intensity of AgNAs@m-SiO_2_ was maintained at a high level, retaining 84.3% of the original signal after 70 days (Figure 5d). A further analysis revealed that by day 35, the enhancement effect of the AgNAs had decreased by 65%. DLS measurements indicated a substantial reduction in aggregate size from approximately 1000 nm to ~130 nm (Figure S3), signifying a transition from multimers to predominantly dimers and dispersed particles, thereby reducing hotspot density. In contrast, AgNAs@m-SiO_2_ maintained a more stable size distribution. Moreover, the zeta potential of the AgNAs dropped to −10.2 mV by day 35, indicating a decrease in the surface charge (Figure S4), which likely reduced the electrostatic adsorption of probe molecules and, consequently, weakened the SERS signal. In contrast, the charge of the AgNAs@m-SiO_2_ remained relatively stable, likely due to the presence of CTAB as a surface ligand. Additionally, a separate long-term storage stability experiment using crystalline violet (CV) for the SERS analysis (Figure S5), with measurements taken every 8 days, is provided in the Supporting Information. The enhancement effect of uncoated AgNAs for CV gradually declined, with a sharp signal drop observed after 40 days. In contrast, AgNAs@m-SiO_2_ exhibited greater stability over 56 days, though a slight decrease was also noted at the 40-day mark.

These results collectively demonstrate that the m-SiO_2_ coating effectively inhibits the depolymerization process of AgNAs, thereby significantly enhancing their long-term stability and improving SERS performance. This underscores the potential of m-SiO_2_-coated AgNAs as a reliable platform for analytical applications requiring sustained sensitivity and durability.

### 3.4. SERS Sensitivity of AgNAs@m-SiO_2_

In order to investigate the SERS detection performance of the prepared substrates, MG, a typical dye molecule and antimicrobial agent, was used as a model for SERS determination at different concentrations (10^−4^ M~10^−7^ M), and linear regression of the relationship between the signal intensity and the concentration was carried out by a logarithmic coordinate system (Figure 6). The regression equation was of the following form:

lgY = 1.0476lgX + 8.7930

where Y is the signal strength, and X is the MG concentration. Based on the signal intensity data and background noise standard deviation (3σ rule), the limit of detection (LOD) for MG was determined to be 3.60 × 10^−^^8^ M (EF = 1.22 × 10^4^), demonstrating the exceptional sensitivity of this SERS substrate. This has some discrepancy with the electric field enhancement factor, which we believe may be due to the combined effect of shell thickness as well as surface charge changes. Furthermore, a strong linear correlation (R^2^ = 0.9972) between the SERS signal intensity and the MG concentration was observed over the range of 10^−4^ M to 10^−7^ M, highlighting its reliability for quantitative analyses. These results collectively underscore the potential of this substrate for practical applications in environmental pollution monitoring and related fields.

### 3.5. Real Sample Testing

**Figure 7:**
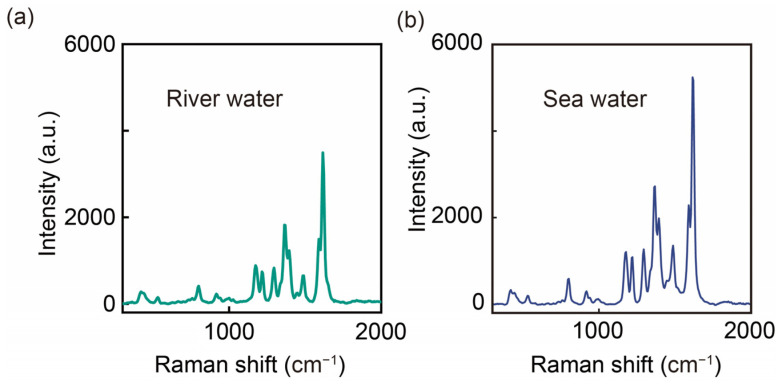
SERS spectra of actual samples containing MG (10^−5^ M): (**a**) river water; (**b**) seawater.

In order to verify the suitability of AgNAs@m-SiO_2_ in the environment, we challenged the suitability of the substrates in complex aqueous media by adding the MGs to real water samples (fresh water from the Fenghuangshan Reservoir and seawater near Yantai University) for direct testing. As shown in Figure 7, high-fidelity MG SERS spectra were obtained for both substrates without pretreatment, demonstrating the possible advantages of the materials in complex media: (1) fouling resistance: the m-SiO_2_ shell acts as a protective shield, allowing MG to diffuse through the channels while excluding larger organic interferences; (2) salt resistance: even in seawater with high salt concentration, MG maintains colloidal stability. Salt-induced aggregation, which is common with SERS substrates, is prevented. This may be due to the combined effect of m-SiO_2_ structural protection and CTAB-modified surface stabilization [33].

Although both samples show recognizable MG signals, we can see a slight difference in SERS intensity between the two samples, which may be due to sample matrix effects (e.g., high salt concentration). Although it is difficult to accurately quantify, it is feasible to qualitatively assess the water quality using our method. For accurate characterization, we may need to optimize sample pretreatment methods to aid in SERS detection, such as diluting the sample. AgNAs@m-SiO_2_-based SERS substrates provide a versatile platform for in situ water quality screening. Its performance in high ionic strength seawater in particular demonstrates its suitability for marine pollution monitoring—a challenging environment where most colloidal SERS substrates fail.

## 4. Conclusions

In this study, a novel SERS substrate based on m-SiO_2_-coated silver aggregates (AgNAs) is developed. AgNAs are first prepared using a “freeze-thaw-ultrasonication” method, followed by m-SiO_2_ coating via a CTAB-assisted silanization reaction to form the AgNAs@m-SiO_2_ composite. The m-SiO_2_ effectively prevents the depolymerization of silver aggregates, maintaining high signal stability under ultrasonication and long-term storage conditions. The SERS signal retention of AgNAs@m-SiO_2_ was 91.8% after 50 min of sonication, which was significantly higher than that of the uncoated AgNAs (45%), and after 70 days of long-term storage, the signal retention of AgNAs@m-SiO_2_ was 84.3%, while that of the uncoated control group was only 8% of the initial signal. The substrate allows for the direct characterization and water quality assessment of real water samples (e.g., river water and seawater), demonstrating its potential for monitoring complex environments. This substrate offers a balance of long-term stability and high sensitivity, providing a promising solution for SERS applications in environmental and biological monitoring. Future work can optimize the silica layer’s thickness and pore structure to improve molecule diffusion and explore other nanomaterial synergies for enhanced performance.

## Data Availability

Data are available upon request to the corresponding author.

## Supplementary Materials

The following supporting information can be downloaded at https://www.mdpi.com/article/10.3390/s25061840/s1, Table S1: Reagents used in the experiment; Table S2: Instruments used in the experiment; Figure S1: TEM images of (a) AgNAs after “freeze-thaw”, (b) AgNAs after “freeze-thaw-ultrasonication”, and (c) AgNAs@mSiO_2_; Figure S2: DLS dynamic light scattering map of AgNAs@mSiO_2_; Figure S3: DLS dynamic light scattering plots of AgNAs and AgNAs@m-SiO_2_ in the initial state and on day 35; Figure S4: Zeta potential plots of AgNAs and AgNAs@m-SiO_2_ in the initial state and on day 35; Figure S5: Comparison of SERS enhancement of CV using (a) AgNAs and (b) AgNAs@m-SiO_2_ over 56 days.
