# Supplementary material for: Utilizing Freeze-Thaw-Ultrasonication to Prepare Mesoporous Silica-Encapsulated Colloidal Silver Nanoaggregates with Long-Term Surface-Enhanced Raman Spectroscopy Activity
Source: Sensors (Basel). 2025 Mar 15;25(6):1840. doi: 10.3390/s25061840 (PMC11946070; doi:10.3390/s25061840)
Supplement: Supplementary file 1 [file sensors-25-01840-s001.zip › sensors-3512818-supplementary.pdf]

Supporting Information for

Utilizing Freeze-Thaw-Ultrasonication to prepare mesoporous silica encapsulated colloidal Ag nanoaggregates with long-term SERS activity

Shuoyang Yan [1], Ling Chen [1,*] and Zhiyang Zhang [2,*]

[1] School of Materials Science and Engineering, University of Jinan, Jinan 250022, China; ysyang026@163.com

[2] Engineering Research Center, Shandong Key Laboratory of Coastal Environmental Processes, CAS Key Laboratory of Coastal Environmental Processes and Ecological Remediation, Yantai Institute of Coastal Zone Research, Chinese Academy of Sciences, Yantai 264003, China

* Correspondence: mse_chenl@ujn.edu.cn (L.C.); zyzhang@yic.ac.cn (Z.Z.)

Reagents and Materials.......... 3

Instrument. 4

Figure S1. 5

Figure S2. 5

Figure S3. 6

Figure S4. 6

Figure S5. 7

Reagents and Materials

Table S1. Reagents used in the experiment

Reagent Name	Purity/Specification	CAS number	Manufacturer
Silver nitrate ($AgNO_3$)	Analytical grade	10018461	Sinopharm Chemical Reagent Co.
Sodium hydroxide (NaOH)	Analytical grade	10019762	Sinopharm Chemical Reagent Co.
Malachite green (MG)	Analytical grade	71023544	Sinopharm Chemical Reagent Co.
Crystal violet (CV)	Analytical grade	71012314	Sinopharm Chemical Reagent Co.
Glycerol	≥99.5%	10010618	Sinopharm Chemical Reagent Co.
Tetraethyl orthosilicate (TEOS)	≥99%	80124118	Sinopharm Chemical Reagent Co.
Anhydrous ethanol	≥99.7%	10009265	Sinopharm Chemical Reagent Co.
Sodium citrate	≥99%	6132-04-3	Aladdin Reagent Co.
Cetyltrimethylammonium chloride (CTAB)	≥99%	57-09-0	Aladdin Reagent Co.
Deionized water (18.2 $M\Omega \cdot cm^{-1}$)	Ultrapure water	——	Thermo Scientific (Avidity Ultrapure Water System)

Instrument.

Table S2. Instruments used in the experiment

Instrument Name	Manufacturer	Model/Specification
UV/Vis/NIR Spectrophotometer	Thermo Scientific, USA	NanoDrop 2000/2000C
Raman Microscope	Thermo Scientific, USA	DXR Raman Microscope
Dynamic Light Scattering (DLS) & Zeta Potential Analyzer	Malvern Instruments, U.K.	Zetasizer NanoZS90
Transmission Electron Microscope (TEM)	Thermo Scientific, USA	Talos F200XG2
Constant Temperature Magnetic Stirrer	Zhengzhou Great Wall Science and Trade Co., Ltd.	DF-101Z
Medical Centrifuge	Hunan Xiangyi Laboratory Instrument Development Co., Ltd.	High-speed centrifuge
Ultrasonic Cleaning Machine	Kunshan Ultrasonic Instrument Co., Ltd.	KQ3200
Glass Capillary Tubes (inner diameter 0.9-1.1 mm)	Instrument Factory of West China Medical University	Custom-made
Three-necked Flask, Beaker, Thermometer	Local Suppliers	Standard laboratory glassware

Ultrapure Water System	Beijing Zeping Technology Agency	Avidity Ultrapure Water Machine

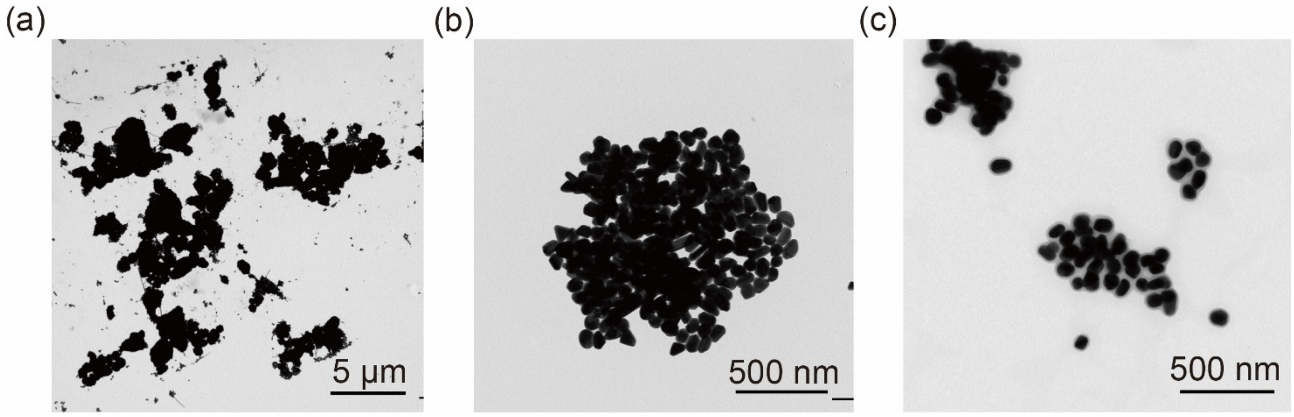

Figure S1. TEM images of (a) AgNAs after “freeze-thaw”, (b) AgNAs after “freeze-thaw-ultrasonication” and (c) AgNAs@mSiO_2.

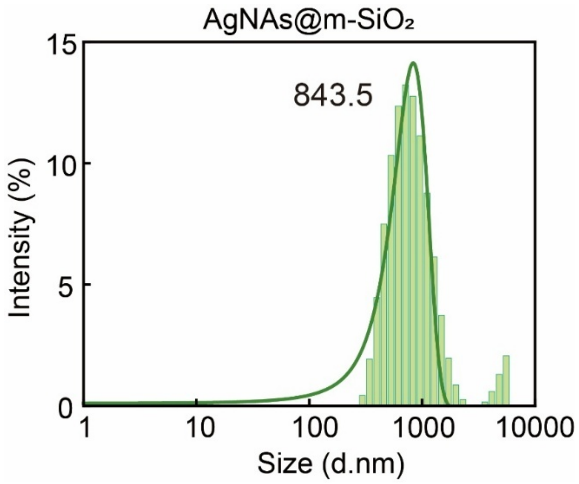

Figure S2. DLS dynamic light scattering map of AgNAs@mSiO_2.

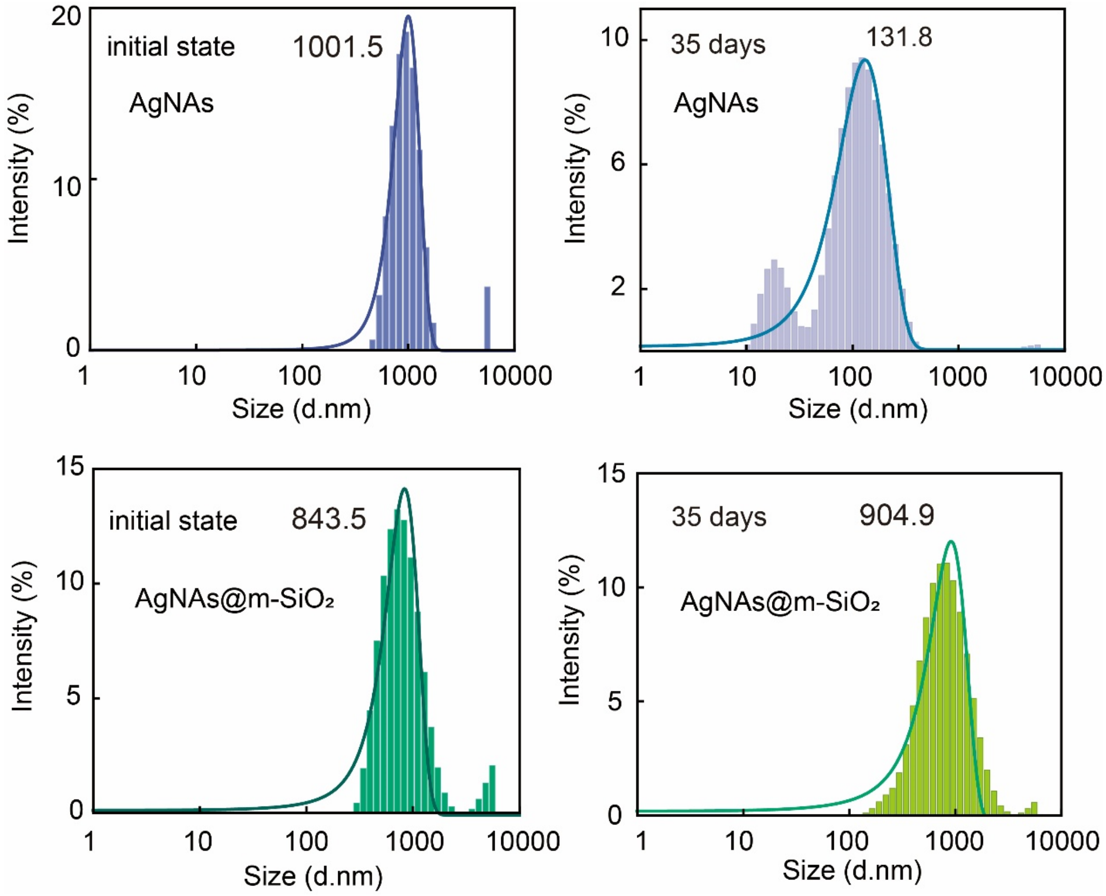

Figure S3. DLS dynamic light scattering plots of AgNAs and AgNAs@m-SiO_2 in the initial state and on day 35.

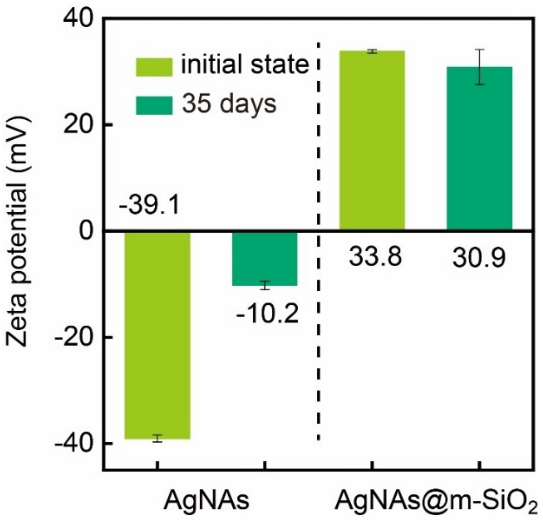

Figure S4. Zeta potential plots of AgNAs and AgNAs@m-SiO_2 in the initial state and on day 35.

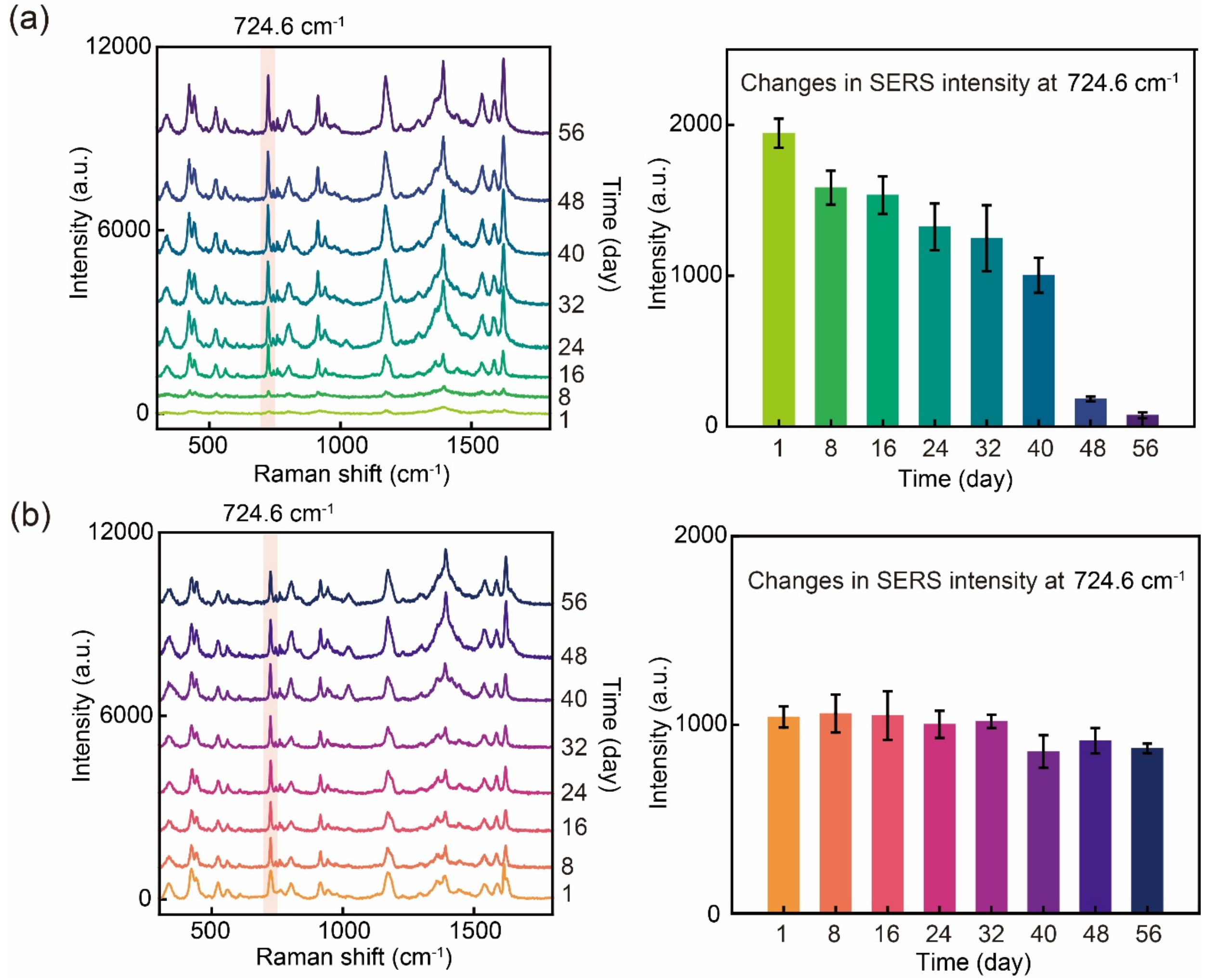

Figure S5. Comparison of SERS enhancement of crystal violet (CV) using (a) AgNAs and (b) AgNAs@m-SiO_2 over 56 days.
